# Novel Traits, Flower Symmetry, and Transcriptional Autoregulation: New Hypotheses From Bioinformatic and Experimental Data

**DOI:** 10.3389/fpls.2018.01561

**Published:** 2018-10-26

**Authors:** Aniket Sengupta, Lena C. Hileman

**Affiliations:** The Hileman Lab, Department of Ecology and Evolutionary Biology, The University of Kansas, Lawrence, KS, United States

**Keywords:** CYCLOIDEA, evolution, flower development, symmetry, transcriptional autoregulation

## Abstract

A common feature in developmental networks is the autoregulation of transcription factors which, in turn, positively or negatively regulate additional genes critical for developmental patterning. When a transcription factor regulates its own expression by binding to *cis*-regulatory sites in its gene, the regulation is direct transcriptional autoregulation (DTA). Indirect transcriptional autoregulation (ITA) involves regulation by proteins expressed downstream of the target transcription factor. We review evidence for a hypothesized role of DTA in the evolution and development of novel flowering plant phenotypes. We additionally provide new bioinformatic and experimental analyses that support a role for transcriptional autoregulation in the evolution of flower symmetry. We find that 5′ upstream non-coding regions are significantly enriched for predicted autoregulatory sites in Lamiales *CYCLOIDEA* genes—an upstream regulator of flower monosymmetry. This suggests a possible correlation between autoregulation of *CYCLOIDEA* and the origin of monosymmetric flowers near the base of Lamiales, a pattern that may be correlated with independently derived monosymmetry across eudicot lineages. We find additional evidence for transcriptional autoregulation in the flower symmetry program, and report that *Antirrhinum DRIF2* may undergo ITA. In light of existing data and new data presented here, we hypothesize how *cis*-acting autoregulatory sites originate, and find evidence that such sites (and DTA) can arise subsequent to the evolution of a novel phenotype.

## Introduction

**FIGURE 1:**
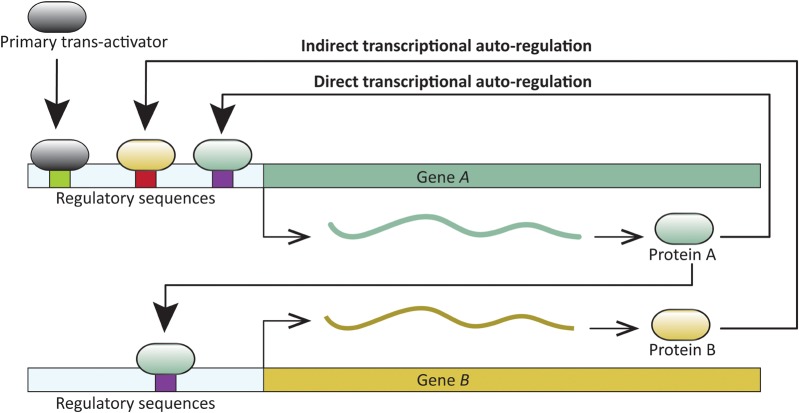
Schematic representation of direct and indirect transcriptional autoregulation (DTA and ITA, respectively) involving two transcription factors. Gene A undergoes DTA and also regulates transcription of gene B. In turn, Gene A undergoes ITA when B regulates its transcription.

A common feature in developmental networks is the autoregulation of transcription factors which, in turn, positively or negatively regulate additional genes critical for developmental patterning. A *trans*-acting protein is considered transcriptionally autoregulated when the protein itself, or downstream factors, modulate its expression. Transcriptional autoregulation can be either direct, or indirect. In direct transcriptional autoregulation (DTA), a protein binds to *cis*-regulatory sites in its gene and modulates expression. Indirect transcriptional autoregulation (ITA) involves regulation by proteins expressed downstream of the target transcription factor (Figure 1). Both DTA and ITA have the potential to enter run-away positive feedback processes. Expression of such genes is likely reduced or stabilized by additional regulatory factors. Transcription factor autoregulation is widespread. For example, at least 40% of transcription factors in *Escherichia coli* are autoregulated (Rosenfeld et al., 2002), and similar direct and indirect autoregulation has been reported across the tree of life—in viruses, prokaryotes, and eukaryotes (for example, Hochschild, 2002; Martínez-Antonio and Collado-Vides, 2003; Holloway et al., 2011; Tao et al., 2012; Gallo-Ebert et al., 2013; and reviewed in Bateman, 1998; Crews and Pearson, 2009), including those with complex development (for example, Cripps et al., 2004; Holloway et al., 2011; Ye et al., 2016). DTA has been demonstrated in processes as diverse, and crucial as the origin of certain cancers (Pasqualucci et al., 2003), and the onset of flowering (Tao et al., 2012).

The widespread occurrence of transcription factor autoregulation suggests a beneficial role in the function and evolution of genetic programs. Here, we provide a review of evidence for DTA in key flowering plant developmental programs. We provide new data supporting the hypothesis that DTA facilitated the evolution of flower monosymmetry in Lamiales. Together these data provide compelling evidence for the hypothesis that DTA plays a role in facilitating the evolution of novelty.

## Advantages of Autoregulation

Several models suggest that autoregulation, especially DTA, can maintain a steady level of expression independent of other factors. If so, genes that are more likely to be autoregulated should be those that experience fleeting regulatory signals, or are positioned upstream in genetic regulatory networks with crucial developmental functions (Crews and Pearson, 2009; Singh and Hespanha, 2009). For example, several transcription factors involved in antibiotic resistance are reported to be autoregulated, resistance being a crucial phenotype (Hoot et al., 2010; Hervay et al., 2011). Similarly, entering or exiting lytic and lysogenic stages is a key developmental decision in lambda bacteriophages, and this decision is partly controlled by the autoregulation of a transcription factor, CI (Hochschild, 2002). The prediction that transcription factors upstream in regulatory networks are more likely to undergo autoregulation has been tested in the model eukaryote yeast, *Saccharomyces* *cerevisiae*. In yeasts, where all possible transcription factor interactions have either been tested or predicted, master regulatory genes are significantly more likely to experience autoregulation than are other regulators (Odom et al., 2006). Similarly, five out of six master regulatory genes in human hepatocytes bind to their own promoters, i.e., undergo DTA (Odom et al., 2006).

How regulatory networks define stable phenotypes is an important question in evolution and development. Simulations of developmental network evolution suggest that autoregulated genes are more robust when faced with random mutations and environmental perturbation (Pinho et al., 2014). The model that DTA stabilizes expression by reducing system noise has been tested in the gene *hunchback* in *Drosophila melanogaster*. Models where the HOX transcription factor Hunchback binds to the *hunchback* promoter (i.e., *hunchback* undergoes DTA) predict less promoter binding-unbinding noise, making the system more robust (Holloway et al., 2011). Experimental work in *hunchback* mutants whose protein cannot bind to DNA (hence, cannot undergo DTA) supports this prediction (Holloway et al., 2011).

In addition to enhancing system robustness, autoregulation provides a mechanism for maintaining expression through key stages of development (reviewed below) that are potentially critical for patterning phenotype. However, the developmental role of DTA has only been tested by mutational studies in a handful of cases. To determine the role of transcription factor DTA, the direct binding between the protein product of a gene and that gene’s *cis-*regulatory DNA can be either intensified or weakened through direct DNA manipulation. For example, addition or deletion of *cis-*regulatory self-binding sites can be used to test for the specific developmental role of DTA within a given species (Espley et al., 2009; Tao et al., 2012; Gallo-Ebert et al., 2013). A complementary, but more difficult approach is to alter transcription factor peptide sequence by mutagenesis in order to modify affinity toward the self-binding sites, e.g., in the *hunchback* exampled discussed above (Holloway et al., 2011). In some model systems, it is possible to repress activity of a transcription factor by overexpressing a dominant chimeric version of the peptide with a repressor domain added to the carboxy-terminus. The chimeric protein can repress the function of the native transcription factor by competitive inhibition (for example, Hiratsu et al., 2003; Koyama et al., 2010). Recent advances in CRISPR/Cas9 gene-editing technologies (Ma et al., 2016) will certainly facilitate exploration of DTA function, at least in model species.

## Review of Dta in Flowering Plant Developmental Evolution

**FIGURE 2:**
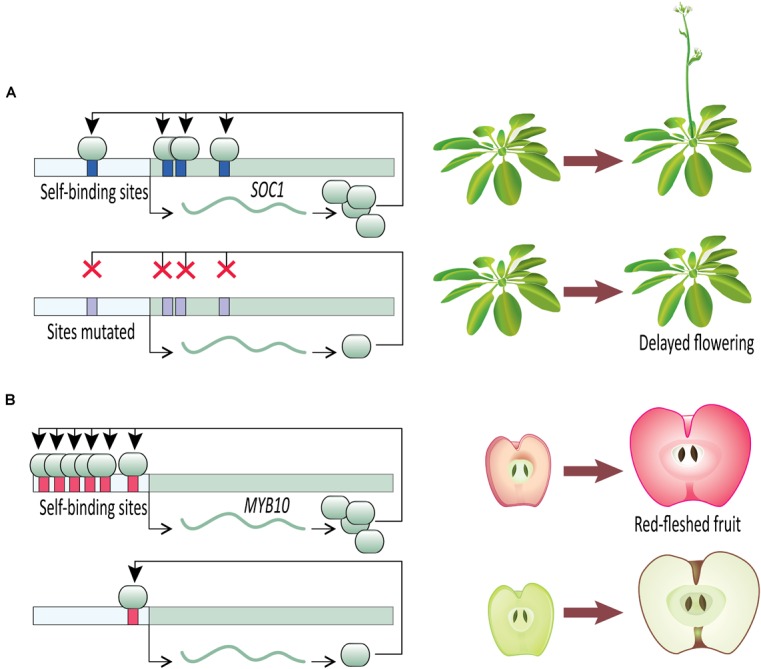
Direct transcriptional autoregulation (DTA) in novel plant phenotypes. **(A)** Disrupting DTA by removal of *cis*-acting autoregulatory sites in *Arabidopsis* *SOC1* delays onset of flowering. **(B)** The number of autoregulatory sites in apple *MYB10* *cis-*regulatory sequence is correlated with fruit flesh color.

Once an initial signal for activation of gene expression has been received, a transcription factor capable of DTA can contribute to swift developmental decisions. A clear example comes from work on the developmental transition to flowering (Figure 2A). Flowering time is a key life-history transition in plant development, intimately tied to environmental cues and aging in order to ensure reproductive success (reviewed in Ó’Maoiléidigh et al., 2014). In *Arabidopsis thaliana*, the transition from vegetative to reproductive development is regulated in part by a MADS-box transcription factor, SUPPRESSOR OF OVEREXPRESSION OF CONSTANS 1 (SOC1). SOC1 undergoes DTA through the binding of SOC1 protein to four *cis*-regulatory *CArG-box* self-binding sites close to the SOC1 transcription start site (Tao et al., 2012). The flowering transition is significantly delayed in the insertional mutant *soc1-2* which carries a loss-of-function mutation in the coding sequence of *SOC1*. The delayed flowering phenotype is largely rescued when *soc1-2* lines are transformed with a wild type *SOC1* allele (including the wild type promoter). This mutant-rescue system with known self-binding sites in the *SOC1* promoter creates an elegant system for testing the specific role of *SOC1* DTA in establishing tight control of the flowering time phenotype. In heterozygous rescue lines where the self-binding sites in the transgenic allele have been mutated by substituting nucleotides at the first two and last two positions of the *CArG-box* binding site, flowering is delayed (Tao et al., 2012). This suggests that the DTA of SOC1 has a key role in transition to flowering. Tao et al. (2012) provide further evidence of SOC1 autoregulation using an estradiol-inducible expression system. Estradiol-induction allows tight control over transgenic protein entering the nucleus and functioning as a transcription factor. Within 2 h of estradiol-induction of transgenic SOC1, expression of endogenous *SOC1* tripled in comparison to a control. This rapid increase in *SOC1* expression after releasing transgenic SOC1 protein to the nucleus suggests SOC1 plays a direct role in its own upregulation. Together, these SOC1 experiments in *Arabidopsis* provide clear evidence that once induced, a transcription factor undergoing DTA can rapidly increase its expression level to swiftly respond to a signal and affect developmental outcomes.

Sustained, stable, and high expression is likely key to defining complex phenotypes. Other than increasing the expression level at a certain point during development (as described in SOC1 above), DTA would provide selective advantage if it could sustain the expression for an extended time through consecutive developmental events. A way to test this would be to determine how expression changes when homologous autoregulatory and non-autoregulatory sites between a pair of recently diverged paralogs are swapped. *Arabidopsis* APETALA1 (AtAP1) and CAULIFLOWER (AtCAL) are two recently duplicated paralogs (Wang et al., 2012) and this system was employed by Ye et al. (2016) to test the role of DTA for sustaining expression in developmental patterning. AtAP1 defines sepal development, and Ye et al. (2016) found that strong expression of *AtAP1* is initiated in floral meristems, and that the expression continues to near-mature flower stages (stage-12). AtAP1 also undergoes DTA wherein it binds to a *CArG*-*box* located in its *cis*-regulatory region and activates *AtAP1* transcription. On the other hand, *AtCAL* does not undergo DTA, is expressed at a low level in early stage flowers, with the expression vanishing soon after stage-4 (Ye et al., 2016). In an elegant system, Ye et al. (2016) generated β-glucuronidase (GUS) reporter-constructs driven by *AtAP1* and *AtCAL* promoter regions. When the *CArG*-*box* in the *GUS* reporter construct with the *AtAP1* promoter was replaced with the homologous non-autoregulatory nucleotides from the *AtCAL* promoter, two changes occurred. First, the overall expression level of *GUS* dropped, and second, the expression duration was shortened, approximating that of *AtCAL* in wild type plants. On the contrary, when *GUS* was placed under the control of an *AtCAL* promoter whose non-autoregulatory nucleotides had been replaced with the homologous *CArG*-*box* from the *AtAP1* promoter, *GUS* expression level increased and extended to near-mature stage flowers. This suggests that DTA of *AtAP1* not only has a role in maintaining high expression levels compared to the non-autoregulated paralog, but has a critical role in sustaining the expression for an extended period. This study did not directly test the role of *AtAP1* DTA, its loss or acquisition, in defining phenotype. However, direct evidence for acquisition or loss of DTA on the evolution of a novel phenotype comes from domesticated apples.

*Malus domestica* (domesticated apple) provides compelling evidence for the importance of DTA on phenotypic outcomes (Figure 2B). The color of fruit flesh in many domesticated apple varieties ranges from white to red. Variation in fruit color is regulated by the transcription factor MYB10, which upregulates anthocyanin expression, especially cyanidin-3-galactoside (Espley et al., 2007, 2009, 2013). Anthocyanin-regulating MYBs have been reported from a wide variety of angiosperm species (reviewed in Lin-Wang et al., 2010), including *Malus* (Espley et al., 2009), *Prunus* (Starkeviè et al., 2015), *Myrica* (Niu et al., 2010), *Arabidopsis* (Gonzalez et al., 2008), and *Ipomoea* (Mano et al., 2007). *Malus domestica* has two alleles of *MYB10* that are identical in their coding sequences but differ in their promoter sequences. Allele *R1* promoter contains one MBY10 autoregulatory binding site, whereas allele *R6* promoter contains six repeats of the autoregulatory site (Espley et al., 2009). The white-fleshed domestic apple varieties are homozygous for the one-repeat *R1* allele, whereas the red-fleshed varieties are *R1*/*R6* heterozygotes or *R6/R6* homozygotes, which leads to increased anthocyanin production via DTA (Espley et al., 2009). It is not clear which allele is ancestral in domesticated apples. Of the four *Malus* species that contributed to the domesticated apple genome (Cornille et al., 2014), *M. sieversii* can be either *R6*/*R6* (Espley et al., 2009; Lin-Wang et al., 2010) or *R1*/*R6* (Espley et al., 2009; van Nocker et al., 2012), and *M. baccata* is *R1*/*R1* (van Nocker et al., 2012). Of the other species in the genus *Malus* tested for *MYB10* promoter sequence, all but one have the *R1*/*R1* genotype (van Nocker et al., 2012).

Though it is not clear whether *R1*/*R1* (white flesh) or *R6*/*R6* (red flesh) is ancestral in the genus *Malus*, it is clear from studies in domesticated apple that changes to fruit flesh color are regulated by addition or loss of autoregulatory sites in the *MYB10* promoter. The evidence from flesh coloration in apples suggests an interesting possibility. Self-activating loops of DTA can serve as easy modules for evolving elevated or reduced gene expression levels. Such evolutionary shifts in gene expression have potentially adaptive developmental consequences accompanied by minimal pleiotropy. Genes, including transcription factors, are often regulated by *trans*-activators that bind to the *cis*-acting elements in the regulatory region of the target gene. Theoretically, these target genes can be upregulated in three ways: adding more *cis*-regulatory sites recognized by either the existing or novel *trans*-activators, upregulating the expression of the existing *trans*-activators, or acquiring new (or additional) self-binding sites in the promoter region. Addition of *cis*-regulatory sites recognized by *trans*-activators can be ineffective if the expression level of the *trans*-activator is limiting. Additionally, increasing the expression level of the *trans*-activator can have pleiotropic consequences. However, acquiring new (or additional) *cis*-regulatory self-binding sites can lead to increased expression of the target gene while bypassing the limitations associated with *trans*-activation. Similarly, reduced expression levels can evolve with minimal pleiotropic consequences through the loss of existing autoregulatory sites.

The evidence from SOC1, AtAP1, and MYB10 provide insight into why genes involved in defining novel phenotypes are likely to undergo DTA. Autoregulatory loops can serve as a quick developmental switch that can rapidly respond to an inbound signal, they can provide high expression levels, and extend that expression through consecutive developmental events. Lastly, DTA can act as a module that can be used to evolve increased or decreased expression with minimal pleiotropic effect, allowing the evolution of novel phenotypes that require such directional changes in protein levels. Quick evolutionary shifts in developmental function of paralogs and divergent alleles can therefore occur through gain or loss of DTA, most likely through gain or amplification of self-binding sites in *cis-*regulatory sequences of focal genes.

## Evidence for Dta in Flower Symmetry Evolution

An emerging system for studying the role of DTA in both development and evolution is flower symmetry. DTA has been implicated in the control of monosymmetry (bilateral symmetry; zygomorphy) (Yang et al., 2012), and may represent a critical step for the evolution of this floral novelty. Monosymmetric flowers are considered a key innovation defining flower form in many species-rich flowering plant lineages including Lamiales, asterids, legumes, and orchids (Sargent, 2004; Vamosi and Vamosi, 2010). Therefore, assessing the role of DTA in the development of flower monosymmetry may provide critical insights into patterns of gene network modification that facilitate novel trait evolution. Below, we review the genetic control of monosymmetry in Lamiales alongside the evidence for DTA. We test for previously unreported regulatory interactions in the *Antirrhinum majus* flower symmetry program, as well as the potential for DTA in a major radiation of taxa with primarily monosymmetric flowers, the Lamiales. Lastly, we comment on possible wide-spread DTA in repeated origins of monosymmetry across flowering plants.

**FIGURE 3:**
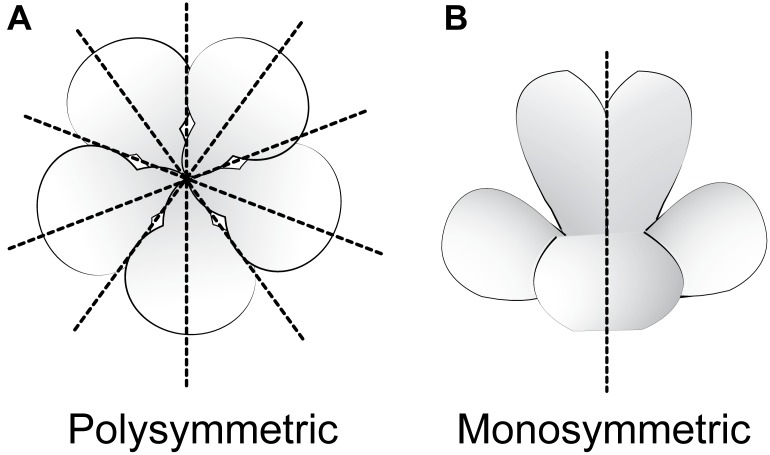
Major types of flower symmetry shown with hypothetical flowers. **(A)** Polysymmetric (radially symmetric, actinomorphic) flower. **(B)** Monosymmetric (bilaterally symmetric, zygomorphic) flower.

Flowering plants are ancestrally polysymmetric (radially symmetric; actinomorphic; Figure 3) (Sauquet et al., 2017). Evolutionary shifts away from polysymmetry include asymmetry (no axis of flower symmetry) and disymmetry (two non-equivalent axes of flower symmetry), but monosymmetry (a single axis of flower mirror-image symmetry; Figure 3) is by far the most common form of non-radial symmetry in flowering plants. Monosymmetric flowers have evolved at least 130 times independently during flowering plant diversification (Reyes et al., 2016). The role of floral symmetry in pollination was recognized as early as 1793 by Sprengel in his monumental German work *Das entdeckte Geheimniss der Natur im Bau und in der Befruchtung der Blumen* (reviewed in Neal et al., 1998; Endress, 1999; Fenster et al., 2004, 2009). Monosymmetric flowers are often associated with specialized pollination by animals (Kampny, 1995; reviewed in Neal et al., 1998), rarely in wind pollinated species (Yuan et al., 2009), and transitions to monosymmetry are strongly associated with increased speciation rates (Sargent, 2004; O’Meara et al., 2016).

**FIGURE 4:**
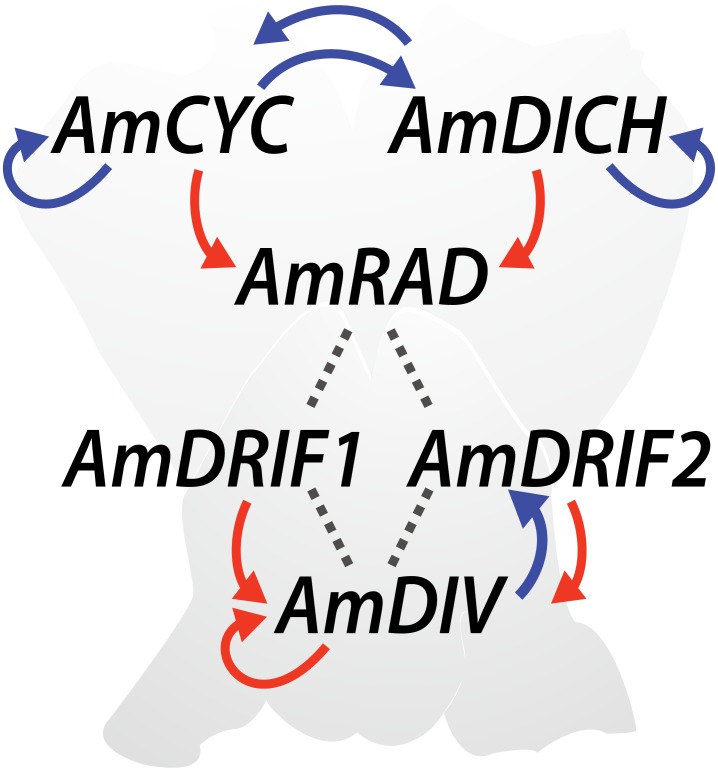
Regulatory mechanisms involved in *Antirrhinum majus* flower symmetry. Previously reported transcriptional regulation (red arrows), transcriptional regulation predicted in this study (blue arrows), and previously reported protein-protein interactions (dotted lines) are shown.

The genetics of monosymmetry is best understood in the model species *A. majus* (snapdragon, Lamiales). The flowers of *A. majus* have two distinct morphological regions—the dorsal (top; adaxial) side, and the ventral (bottom; abaxial) side (Figure 4). Monosymmetry of *A. majus* flowers along the dorso-ventral axis is defined by a competitive interaction involving TCP and MYB transcription factors. TCP (TEOSINTE BRANCHED1, CYCLOIDEA, and PROLIFERATING CELL FACTORS) and MYB (first described from avian myeloblastosis virus) proteins are found as large gene families in flowering plants (Yanhui et al., 2006; Martín-Trillo and Cubas, 2010) and play diverse roles in aspects of vegetative and reproductive developmental patterning (Martín-Trillo and Cubas, 2010; Ambawat et al., 2013; Parapunova et al., 2014). The dorsal side of *A. majus* flowers is defined by the combined action of two recently duplicated TCP paralogs, CYCLOIDEA (AmCYC) and DICHOTOMA (AmDICH) (Luo et al., 1996, 1999; Hileman and Baum, 2003; Corley et al., 2005). These two transcription factors define dorsal flower morphology partly by activating the transcription of a downstream MYB protein, RADIALIS (AmRAD; Figure 4) (Corley et al., 2005). AmRAD post-translationally negatively regulates another MYB protein, DIVARICATA (AmDIV), which defines ventral flower morphology. Through this negative interaction, AmRAD excludes the ventral flower identity specified by AmDIV from the dorsal side of the developing *A. majus* flower (Figure 4). Specifically, AmRAD and AmDIV compete for interaction with two MYB-family protein partners called DIV and RAD Interacting Factors 1 and 2 (AmDRIF1 and AmDRIF2) (Almeida et al., 1997; Galego and Almeida, 2002; Corley et al., 2005; Raimundo et al., 2013). AmDIV requires protein-protein interaction with AmDRIF1 or 2 to function as a transcription factor and upregulate its own transcription, as well as to regulate downstream targets (Figure 4) (Perez-Rodriguez et al., 2005; Raimundo et al., 2013). In the dorsal flower domain, AmRAD outcompetes AmDIV for interaction with AmDRIF1/2, preventing accumulation of AmDIV protein (Raimundo et al., 2013).

Because flower monosymmetry has evolved multiple times, a considerable amount of effort has gone into testing whether elements of the *A. majus* symmetry program function to specify dorso-ventral differentiation in other flowering plant lineages. Interestingly, all monosymmetric species tested at a molecular level so far show evidence that a TCP-based regulatory network is likely involved in differentiation along the dorso-ventral flower axis. These studies span eudicot and monocot lineages and primarily, but not exclusively, show a pattern of dorsal-specific floral expression of *TCP* homologs (for example, Citerne et al., 2003, 2010; Busch and Zachgo, 2007; Wang et al., 2008; Yuan et al., 2009; Bartlett and Specht, 2011; Howarth et al., 2011; Chapman et al., 2012; Preston and Hileman, 2012; Damerval et al., 2013; and reviewed in Hileman, 2014). In core eudicots, there are three lineages of *CYCLOIDEA* (*CYC*)-like TCP genes resulting from two rounds of duplication near the origin of core eudicots: the *CYC1*-, *CYC2*-, and *CYC3*-lineages (Howarth and Donoghue, 2006; Citerne et al., 2013). *AmCYC* and *AmDICH* belong to the *CYC2*-lineage, and in an interesting pattern, all TCP genes implicated in floral monosymmetry in core eudicots belong to the same *CYC2*-lineage (Howarth and Donoghue, 2006; Citerne et al., 2010; and reviewed in Hileman, 2014). How these orthologous genes were recruited convergently during the multiple evolutionary origins of floral monosymmetry, from an as yet unclear function in species with ancestral polysymmetry, remains an open question.

Detailed developmental studies in *A. majus* have provided key insights into the regulatory interactions that shape flower monosymmetry, and *A. majus* as a model represents a species-rich lineage of flowering plants, Lamiales. Monosymmetry evolved early in Lamiales diversification (Zhong and Kellogg, 2015; Reyes et al., 2016), and developmental genetic studies in additional Lamiales species provide further insight into the regulatory network that shapes bilateral flower symmetry across the entire lineage. Notably, detailed expression and functional studies of *CYC, RAD* and *DIV* orthologs in Gesneriaceae, a sister lineage to the bulk of Lamiales species diversity, have contributed to a fuller understanding of regulatory interactions that shape Lamiales flower monosymmetry (Citerne et al., 2000; Smith et al., 2004; Gao et al., 2008; Zhou et al., 2008; Yang et al., 2010, 2012; Liu et al., 2014a). From studies in *A. majus* (Plantaginaceae) and *Primulina heterotricha* (syn. *Chirita heterotricha;* Gesneriaceae), there is strong evidence that at least two components of the flower symmetry network undergo DTA–*DIV* and *CYC* (Figure 4).

As mentioned above, AmDIV forms heterodimers with AmDRIF1 and 2 to specify ventral flower identity in *A. majus* (Raimundo et al., 2013). AmDIV-AmDRIF dimers bind to a consensus sequence that includes the conserved *I-box* motif, 5′-GATAAG-3′ located 2596 bp upstream of the *AmDIV* transcription start site (Raimundo et al., 2013), providing compelling evidence that AmDIV is involved in an autoregulatory loop. Autoregulation of *DIV* orthologs has not been tested outside of *A. majus*. In *P. heterotricha*, peloric (radialized) forms due to flower ventralization have reduced expression levels of *CYC* orthologs, *PhCYC1C* and *PhCYC1D* (Yang et al., 2012), presenting strong evidence that these two genes define dorsal identity of monosymmetric *P. heterotricha* flowers. Experimental evidence suggests that PhCYC1 and PhCYC2 undergo DTA; PhCYC1 and PhCYC2 proteins bind to the consensus TCP-binding sequence 5′-GGNCCC-3′ in the putative promoter regions of both *PhCYC1* and *PhCYC2* (Yang et al., 2010, 2012). Autoregulation of *CYC* orthologs has not been tested outside of *P. heterotricha*.

These initial insights from *A. majus* and *P. heterotricha* lead to a set of important evolutionary questions. Is autoregulation of *CYC* orthologs conserved across Lamiales? And has a pattern of autoregulation repeatedly evolved in *CYC2*-lineage orthologs from lineages with independently derived monosymmetric flowers? This second question is especially compelling given that *CYC2*-lineage ortholog expression is expected to persist from early through later stages of flower development in order to specify asymmetric morphological differentiation along the dorso-ventral floral axis in lineages with flower monosymmetry.

### Methods: Evidence for DTA in Flower Symmetry Evolution

#### Homolog Predictions and Phylogenetic Analyses

*AmCYC, AmDICH, AmRAD*, and *AmDIV* orthologs were identified from published sources and online databases by tBLASTx (Altschul et al., 1990). The gene names/identifiers and sources are listed in Supplementary Tables 1, 2. Gene identifiers are also included with terminal genes on the phylogenies (Supplementary Figures 1, 2). A subset of included genes were available as full-length coding sequences from public databases. A subset of included genes were available as partial coding sequences from public databases. For partial coding sequences from species with available genome data, we predicted the full-length coding sequences either manually by aligning to previously reported homologs, or by prediction with AUGUSTUS (Stanke et al., 2004). A subset of included genes were identified by BLAST (Altschul et al., 1990) from annotated genomes. We predicted the coding sequences either manually or with AUGUSTUS when our BLAST searches hit a region in a genome where no or partial genes were predicted. For *Mimulus lewisii* *DIV* and *RAD* homologs, we first BLAST searched the available transcriptome and subsequently mapped the hits to the genome. Two sets of sequences used here were not publicly available, the genes from *Ipomoea lacunosa* whose genome sequence was generously shared by Dr. Mark Rausher (Duke University), and *Mimulus guttatus RADlike1*, which was shared by Dr. Jinshun Zhong (University of Vermont; the sequence was reported in Zhong et al., 2017).

We translationally aligned the coding sequences (omitting the stop codon) of *CYC*-like genes using MAFFT v7.388 (Katoh et al., 2002) in Geneious 10.2.3 (Kearse et al., 2012) with the following parameters: algorithm–auto, scoring matrix–BLOSUM62, gap opening penalty–1.1, offset value–0.124. The entire alignment was used for downstream phylogenetic analyses. The *CYC*-like gene tree was estimated using a Bayesian approach (Metropolis-coupled Markov chain Monte Carlo) in MrBayes 3.2.6 (Ronquist et al., 2012) with uninformative priors for 10 million generations on the online CIPRES portal at https://www.phylo.org (Miller et al., 2010). The core-eudicot *CYC-*like tree was rooted with Rananculales *CYC-*like genes in FigTree^1^.

*DIV-* and *RAD-*like genes were translationally aligned using an approach similar to *CYC*-like genes except for the following: gap opening penalty–1.53, and offset value–0.123. We removed the columns with 70% or more gaps from the alignment, and from the subsequent file used only the conserved first *MYBI* domain and nucleotides immediately 3′ to this domain. *DIV-* and *RAD-*like gene trees were estimated using the same approach as for *CYC*-like genes. Resulting *DIV-* and *RAD-*like trees were mid-point rooted in FigTree^1^. For all sequences included in our phylogenetic analyses, nexus format nucleotide alignment along with the Bayesian parameter block, and the unaligned coding sequences in fasta format available from the Dryad Digital Repository: https://doi.org/10.5061/dryad.tv54037.

#### Consensus TCP and DIV-Binding Site Predictions

We downloaded up to 3 kb non-coding sequence upstream of the transcription start sites of target Lamiales *CYC, RAD*, and *DIV* homologs from corresponding genomes. We downloaded up to 3 kb non-coding sequence upstream of the transcription start sites of representative core eudicot *CYC* homologs from corresponding genomes. All genomic sources are listed in Supplementary Table 1. Within these sequences, we searched for the consensus TCP-binding site 5′-GGNCCC-3′ (Kosugi and Ohashi, 2002; Costa et al., 2005; Yang et al., 2012; Gao et al., 2015) on both strands using Geneious 10.2.3 (Kearse et al., 2012). In *A. majus* only, we searched for the consensus DIV-binding site, 5′-[AGC]GATA[AC][GC][GAC]-3′ (Raimundo et al., 2013) in 3 kb upstream non-coding sequences of the six genes known to be involved in *A. majus* flower symmetry (Figure 4) using Geneious 10.2.3 (Kearse et al., 2012). To determine whether the consensus TCP-binding sites found in the *A. majus* and *M. lewisii* upstream *CYC* homolog sequences were derived from other genomic locations, we used the predicted TCP-binding sites, plus 100 bp on either side, as BLAST queries against the available genomes in Geneious 10.2.3 (Kearse et al., 2012).

#### Analysis of Motif Enrichment

We tested for consensus TCP-binding site enrichment using *Analysis of Motif Enrichment* (AME^2^; McLeay and Bailey, 2010). AME can identify known or user-provided motifs that are relatively enriched in a given set of sequences compared with shuffled versions of those sequences or with user-provided control sequences. AME does not discriminate among motifs based on their locations within the sequences. The following options were selected: sequence scoring method—average odds score, motif enrichment test—rank sum test, and background model—uniform model. We defined the consensus TCP-binding site as 5′-GGNCCC-3′ (Kosugi and Ohashi, 2002; Costa et al., 2005; Yang et al., 2012; Gao et al., 2015), and query sequences as 3 kb upstream of transcription start sites of focal genes, and used shuffled sequences as the control. The upstream non-coding sequences are available in fasta format from the Dryad Digital Repository: https://doi.org/10.5061/dryad.tv54037.

#### Quantitative Reverse-Transcriptase PCR (rt-PCR)

*Antirrhinum majus* wild type (genotype JI 7) and *divaricata* mutants (genotype JI 13) were acquired from John Innes Centre, United Kingdom, under USDA Permit No. P37-16-01034. Five flower buds of the same developmental stage (stage-11, flower bud *ca.* 4.0 mm in length, corolla equal in length to calyx, petal tips white in wild type; Vincent and Coen, 2004) were sampled from each genotype. RNA was extracted using RNeasy plant minikit (Qiagen, Germantown, MD, United States), followed by DNase treatment (TURBO^TM^ DNase, Thermo Fisher Scientific, Waltham, MA, United States), and cDNA synthesis (iScript cDNA Synthesis Kit, Bio-Rad, Hercules, CA, United States). Quantitative rt-PCR was performed on a StepOnePlus^TM^ Real-Time PCR System (Thermo Fisher Scientific) using SYBR^TM^ Select Master Mix (Thermo Fisher Scientific). Quantitative rt-PCR was carried out for three technical replicates for each of five biological replicates per genotype. Expression was normalized against *UBIQUITIN5*. This gene has been reported to have little transcriptional variation across tissue types and developmental stages (Preston and Hileman, 2010). Expression was analyzed by the ΔΔCt method. Significant differences in relative expression between genotypes were determined using two sample *t*-test assuming equal variances in Minitab. The quantitative rt-PCR primers were as follows: AmDRIF1_RT_F4: GCCTTGGATCAAATTTCGGC; AmDRIF1_RT_R4: AGGAAGAATGGAGCTGGCAA; AmDRIF2_RT_F1a: AATGGTCATGGAGAGTGGGG; AmDRIF2_RT_R1:TATAGCTTGCTCCTCTGGGG; AmUBQ5_qPCR_F1: GCGCAAGAAGAAGACCTACAC; AmUBQ5_qPCR_R1: CTTCCTGAGCCTCTGCACTT. Efficiency of PCR was determined using DART (Peirson et al., 2003).

### Results: Evidence for DTA in Flower Symmetry Evolution

#### Predicted TCP- and DIV-Binding Sites in *A. majus* Are Consistent With Known and Hypothesized Transcriptional Regulation

In *A. majus*, we found consensus TCP-binding sites in four of the six genes known to be involved in *A. majus* flower symmetry (Figure 4 and Table 1). *AmCYC* and *AmDICH* had eight and four predicted TCP-binding sites in their upstream non-coding sequences, respectively, and likely regulate their own and each other’s expression. Notably, *AmCYC* DTA has been hypothesized previously (Costa et al., 2005), and the presence of predicted autoregulatory sites in *AmCYC* and *AmDICH* is consistent with the putative auto and cross-regulation of *P. heterotricha PhCYC1C* and *PhCYC1D* (Yang et al., 2012). *AmRAD*, known to be positively regulated by AmCYC and AmDICH (Corley et al., 2005; Costa et al., 2005), had two predicted consensus TCP-binding sites in its upstream non-coding sequence. *AmDIV* and *AmDRIF2* did not have predicted TCP-binding sites in their upstream non-coding sequences, consistent with evidence that they are unlikely to be under direct transcriptional regulation by AmCYC, AmDICH, or any other more distantly related TCP transcription factors.

**Table 1:** Predicted consensus TCP-binding sites in the upstream non-coding sequences of *A. majus* flower symmetry genes.

Gene	Sequence	DNA strand	bp upstream of transcription start
*AmCYC*	**GG**G**CCC**	Sense	2454–2457
	**GG**G**CCC**	Sense	544–549
	**GG**G**CCC**	Anti-sense	544–549
	**GG**G**CCC**	Anti-sense	2452–2457
	**GG**C**CCC**	Sense	2451–2456
	**GG**C**CCC**	Sense	2292–2297
	**GG**C**CCC**	Sense	543–548
	**GG**C**CCC**	Anti-sense	2453–2458
*AmDICH*	**GG**G**CCC**	Sense	1170–1175
	**GG**G**CCC**	Anti-sense	1170–1175
	**GG**C**CCC**	Sense	965–970
	**GG**C**CCC**	Anti-sense	1171–1176
AmRAD Costa et al., 2005	**GG**C**CCC**	Sense	1521–1526
	**GG**C**CCC**	Sense	1489–1494
*AmDRIF1*	**GG**T**CCC**	Anti-sense	2394–2399

*Bases in bold indicate conservation in the consensus binding site. *AmDIV and AmDRIF2* lack consensus TCP-binding sites in their upstream non-coding sequences. Costa et al. (2005) reported TCP-binding sites for *AmRAD* and suggested the presence of autoregulatory sites in the non-coding sequence upstream of AmCYC*.

Consensus TCP-binding sites (plus 100 bp flanking sequence from either side) initially identified in the upstream non-coding sequences of *AmCYC* and *AmDICH* were used to search for similar sites elsewhere in the *A. majus* genome. These searches resulted in only self-hits to *AmCYC* and *Am*DICH upstream non-coding sequences or cross-paralog matches between *AmCYC* and *AmDICH*. This result suggests that these sites evolved *de novo* and not through translocation of existing sites from elsewhere in the genome. Similarly, our search for consensus TCP-binding sites from *M. lewisii* *CYC2-*lineage genes in the *M. lewisii* genome resulted in only self-hits.

We identified two consensus DIV-binding sites in the *AmDIV* upstream non-coding sequence (Table 2), one of which was previously iTA. *AmCYC, AmRAD, AmDRIF1* and *AmDRIF2*, but not *AmDICH*, also had predicted DIV-binding sites in their upstream non-coding sequences (Table 2). It is unlikely that the predicted DIV-binding sites in the upstream non-coding sequences of *AmCYC* or *AmRAD* function for AmDIV binding. This is because AmDIV function is impaired in the presence of AmRAD proteins through competitive inhibition.

**Table 2:** Predicted consensus DIV-binding sites in the upstream non-coding sequences of *A. majus* flower symmetry genes.

Gene	Sequence	DNA strand	bp upstream of transcription start
*AmCYC*	A**GATA**AGG	Anti-sense	329–336
*AmRAD*	A**GATA**ACA	Anti-sense	798–805
	G**GATA**ACG	Anti-sense	1051–1058
	C**GATA**AGA	Anti-sense	2843–2850
AmDIV Raimundo et al., 2013	A**GATA**AGG	Sense	2595–2602
	C**GATA**CCC	Sense	1557–1564
*AmDRIF1*	G**GATA**CGG	Sense	711–718
	A**GATA**AGG	Sense	242–249
	A**GATA**AGC	Anti-sense	505–512
*AmDRIF2*	A**GATA**ACC	Anti-sense	1892–1899

*Bases in bold indicate conservation in the consensus binding site. *AmDICH* lacks consensus DIV-binding sites in upstream non-coding sequences. One of two consensus DIV-binding sites in *AmDIV* was reported by Raimundo et al. (2013)*.

#### Expression Analyses Suggest Additional Autoregulation of DIV in *A. majus*

**FIGURE 5:**
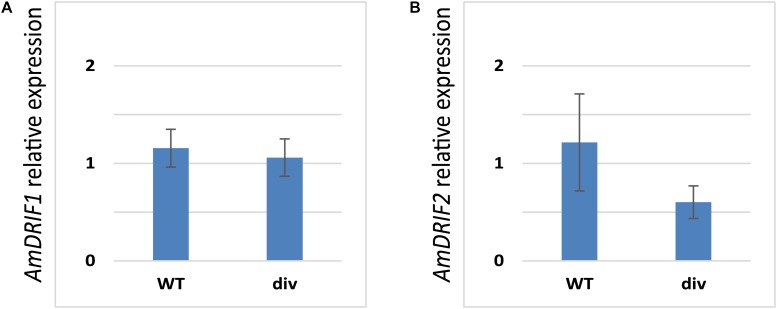
Relative expression of *AmDRIF1* **(A)** and *AmDRIF2* **(B)** in wild type and *divaricata* mutant lines. The expression level of *AmDRIF2* is significantly lower in the div background suggesting that AmDIV positively regulates *AmDRIF2* transcription. The values are mean ± standard deviation.

Given the presence of predicted DIV-binding sites in *AmDRIF1* and *AmDRIF2* upstream non-coding sequences (Table 2), we tested whether *AmDRIF1* and/or *AmDRIF2* expression is significantly altered in the *A. majus div* mutant background compared to wild type. We found that *AmDRIF1*, despite having multiple *DIV* consensus binding sites in its upstream region, was not under either direct or indirect regulation by AmDIV (*p* = 0.453; Figure 5A). *AmDRIF1* may be regulated by a non-DIV MYB transcription factor(s) that binds to the consensus DIV-binding motif. On the other hand, we found significantly lower levels of *AmDRIF2* expression in *div* mutant flower buds compared to wild type (*p* = 0.031; Figure 5B). This suggests that *AmDRIF2* is either directly or indirectly positively regulated by AmDIV. In turn, *AmDIV* is positively regulated by AmDRIF2-AmDIV heterodimers (Raimundo et al., 2013). Therefore, *AmDIV* appears to experience both direct and ITA through interaction of *AmDIV cis-*regulatory sequences with AmDRIF2-AmDIV heterodimers.

#### Putative TCP-Binding Sites Are Enriched in Upstream Non-coding Sequences of Lamiales *CYC2*-Lineage Genes

While no *CYC2*-lineage gene outside *P. heterotricha* has been experimentally tested for DTA, it is possible to infer the potential for DTA by screening for the consensus TCP-binding site, 5′-GGNCCC-3′ (Kosugi and Ohashi, 2002; Yang et al., 2012; Gao et al., 2015), in putative *cis*-regulatory regions of Lamiales *CYC2*-lineage genes. Given that flower monosymmetry is homologous in *P. heterotricha* and *A. majus*, evolving early in the diversification of Lamiales (Zhong and Kellogg, 2015; Reyes et al., 2016), a straight-forward hypothesis is that *CYC2*-lineage DTA evolved early in Lamiales and has been retained in Lamiales lineages with monosymmetric flowers. Under this hypothesis, Lamiales with flower monosymmetry will retain consensus TCP-binding site(s) in putative *CYC2*-lineage *cis*-regulatory sequences. The availability of multiple Lamiales genomes (Supplementary Table 1) allowed us to begin testing the hypothesis that autoregulation is potentially conserved across Lamiales *CYC* orthologs.

We identified orthologs of *AmCYC/AmDICH* (*CYC2-*lineage genes) from genome-sequenced Lamiales plus representative core eudicots (Supplementary Figure 1 and Table 1). We identified orthologs of *AmRAD* and *AmDIV* from genome-sequenced Lamiales plus representative orthologs from sister lineages to Lamiales, Gentianales, and Solanales (Supplementary Figure 2 and Table 2). As with *P. heterotricha* and *A. majus*, recent duplication events lead to paralog complexity for *CYC2*-lineage genes (Supplementary Figure 1). We found that at least one *CYC2*-lineage gene from each core eudicot species had consensus TCP-binding sites(s) in the upstream non-coding sequence (Supplementary Tables 3, 4), with two exceptions. The only *CYC2*-lineage genes in *Vitis vinifera* (Vitales), *CYCLOIDEA-like 2a*, and *Gossypium raimondii* (Malvales), *TCP1*, had no consensus TCP-binding sites in their upstream non-coding sequences.

We found consensus TCP-binding sites in the upstream non-coding sequences of *CYC2*-lineage genes in a wide variety of core eudicots with flowers with mono-, poly-, and dissymmetry (Supplementary Tables 3, 4). However, *prima facia*, the *CYC2-*lineage orthologs from Lamiales appeared to be enriched for consensus TCP-binding sites. We tested for enrichment of consensus TCP-binding sites in the non-coding sequences upstream of Lamiales *CYC2-*lineage genes. Additionally, we tested the upstream non-coding sequences of non-Lamiales core-eudicot *CYC2-*lineage genes, and Lamiales *RAD* and *DIV* orthologs for enrichment in consensus TCP-binding sites. We predict that *RAD* orthologs may show enrichment of the consensus TCP-binding site due to conserved regulation of *RAD* by CYC*-*like transcription factors across Lamiales, but that Lamiales *DIV* orthologs are not likely to be enriched for the consensus TCP-binding site given that there is no previous data indicating regulation of *DIV* orthologs by CYC-like transcription factors or other TCP proteins.

As expected, we found that the upstream non-coding sequences of Lamiales *DIV* orthologs were not significantly enriched for the consensus TCP-binding sites (*p* = 0.517; Table 3), and that the upstream non-coding sequences of Lamiales *RAD* orthologs were significantly enriched for the consensus TCP-binding site (*p* = 0.0406; Table 3). This result is consistent with CYC-like transcription factors acting as regulators of *RAD*, but not *DIV* across Lamiales. Strikingly, we found that the upstream non-coding sequences of *CYC2*-lineage genes in Lamiales were significantly enriched in consensus TCP-binding sites (*p* = 0.0169; Table 3) in-line with the hypothesis that *CYC* autoregulation evolved early in Lamiales, coincident with the evolution of monosymmetric flower, and has been maintained during Lamiales diversification. Notably, this pattern of enrichment appears specific to Lamiales. We tested for similar enrichment of the consensus TCP-binding site in non-Lamiales core eudicot *CYC2-*lineage orthologs and found no evidence for a similar pattern of binding site enrichment (*p* = 0.352; Table 3).

**Table 3:** Results from analysis of motif enrichment (AME) tests for consensus TCP-binding sites in the upstream non-coding sequences of symmetry gene orthologs.

Test sequences (putative *cis*-regulatory regions)	Control sequences	*p*-Value	Genes surveyed	Species surveyed
Lamiales *DIV* orthologs	Shuffled test sequences	0.517	15	9
Lamiales *RAD* orthologs	Shuffled test sequences	**0.0406**	33	9
Lamiales *CYC2* orthologs	Shuffled test sequences	**0.0169**	20	9
Non-Lamiales core eudicot *CYC2* orthologs	Shuffled test sequences	0.352	39	17

*Significant *p*-values (below 0.05) are in bold*.

## Discussion

### Binding Site Enrichment Supports the Hypothesis That DTA of *CYC* Is Associated With the Origin of Flower Monosymmetry in Lamiales

Positive regulation of *RAD* by CYC2-lineage genes for specifying flower monosymmetry is conserved across much of Lamiales (Corley et al., 2005; Zhou et al., 2008; Su et al., 2017). That we find significant enrichment of consensus TCP-binding sites in Lamiales *RAD* upstream non-coding sequences is in-line with conservation of this *CYC-RAD* regulatory module. Strikingly, our data demonstrate that Lamiales *CYC2-*lineage genes are also significantly enriched for consensus TCP-binding sites in upstream non-coding sequences. This supports the hypothesis that the origin of Lamiales flower monosymmetry coincides with the evolution of *CYC2-*lineage DTA. Further empirical studies in emerging Lamiales models (e.g., Liu et al., 2014b; Su et al., 2017) will allow this hypothesis to be tested, as well as the alternative, that *CYC2-*lineage genes undergo transcriptional regulation by other TCP family proteins. As additional eudicot genomes become available, tests for TCP-binding site enrichment can be carried out in other lineages with bilaterally symmetrical flowers for which a role of *CYC2-*lineage genes has been implicated, for example, Fabaceae (Wang et al., 2008; Xu et al., 2013) and Malpighiaceae (Zhang et al., 2010).

### Evaluating the Pan-Eudicot Model for Monosymmetry Involving DTA of *CYC2*-Lineage Genes

**FIGURE 6:**
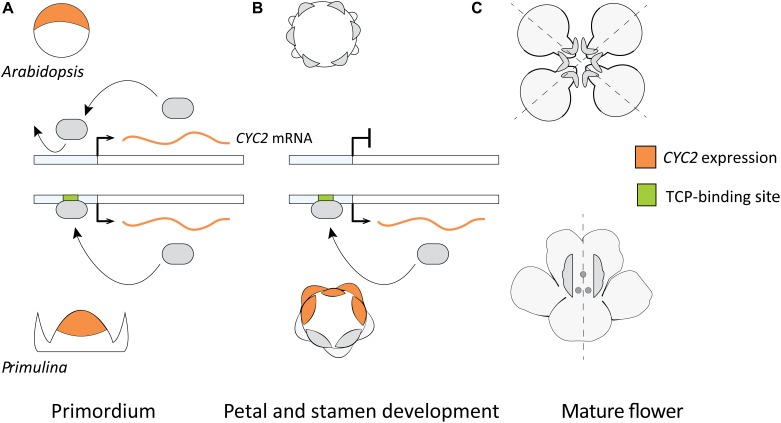
A previously proposed model explaining flower symmetry in *Primulina heterotricha* and *Arabidopsis*. **(A)** *CYC2*-lineage genes are expressed in early stage flower primordia of both *Arabidopsis* (dorsal part) and *P. heterotricha* (apical part). **(B)** Expression in *P. heterotricha* continues by DTA to later stages crucial for defining flower monosymmetry, this is not the case in *Arabidopsis.* **(C)** At anthesis, *P. heterotricha* is monosymmetric, *Arabidopsis* is not.

A model hypothesizing the role of DTA for the parallel origin of monosymmetric flowers across eudicots was put forward by Yang et al. (2012; Figure 6) based on two primary lines of evidence. First, the observed differences in duration of flower specific expression of *CYC2*-lineage genes between species with monosymmetric vs. non-monosymmetric flowers. Second, the reported absence of consensus TCP-binding sites in the upstream non-coding sequences of *CYC2*-lineage genes from non-monosymmetric flowers. Specifically, *Arabidopsis thaliana, Brassica* *rapa, Vitis* *vinifera*, and *Solanum* *lycopersicum* do not have monosymmetric flowers and were reported to lack consensus TCP-binding sites in their *CYC2*-lineage genes compared to *Glycine* *max, Medicago* *trunculata, Mimulus* *guttatus, Primulina* *heterotricha, Oryza* *sativa*, and *Zea mays* (representing three independent origins of monosymmetry) that have consensus TCP-binding sites (Yang et al., 2012).

This model relies heavily on observations from *Arabidopsis* flowers where the expression of the sole *CYC2*-lineage gene (*AtTCP1*) is transiently dorsal-specific and the flowers are non-monosymmetric (Cubas et al., 2001). It is clear that AtTCP1 does not play a critical role in floral organ differentiation in *Arabidopsis*, given no floral-specific DTA or other means by which expression can persist to later stages of flower differentiation. However, the pattern in *Arabidopsis* may not be universal for non-monosymmetric flowers. Closely related monosymmetric and non-monosymmetric Brassicaceae flowers do not exhibit a consistent pattern of early dorsal-specific expression (Busch et al., 2012). Evidence from Brassicaceae suggests that *Arabidopsis*-like dorsal-restricted expression early in flower development is not a pre-requisite for the evolution of flower monosymmetry via DTA. Beyond Brassicaceae, there are examples of ancestrally non-monosymmetric flowers in core-eudicots where expression of *CYC2*-lineage genes is not localized spatially and/or restricted to an early developmental stage. These examples include *Bergia texana* (Elatinaceae) (Zhang et al., 2010), *Viburnum plicatum* (Adoxaceae) (Howarth et al., 2011), and *Solanum lycoperscicum* (Solanaceae, ancestral state ambiguous) (Parapunova et al., 2014), as well as an early-diverging eudicot, *Eschscholzia californica* (Papaveraceae) (Kölsch and Gleissberg, 2006).

Yang et al. (2012) reported a correlation between flower monosymmetry vs. non-monosymmetry and the presence vs. absence of consensus TCP-binding sites in corresponding upstream non-coding sequences of *CYC2*-lineage genes. This contributed to the model for the origin of flower monosymmetry facilitated by the evolution of *CYC2-*lineage DTA. In our expanded sampling we find that consensus TCP-binding sites are present in the upstream non-coding sequences of many *CYC2*-lineage genes across eudicots irrespective of flower symmetry. Yet, in an interesting pattern, all species with independently derived monosymmetric flowers that we investigated (Fabales, Lamiales, Brassicales, Asterales) have at least one *CYC2*-lineage ortholog with a consensus TCP-binding sequence in the upstream non-coding sequences (Supplementary Tables 3, 4). On the other hand, many species with non-monosymmetric flowers also have at least one *CYC2*-lineage ortholog with a consensus TCP-binding sequence in their upstream non-coding sequences (Supplementary Tables 3, 4). Notably, we find that the sole *CYC2*-lineage gene in *Arabidopsis* (*AtTCP1*), and a second *CYC2-*lineage gene in tomato that was not included in Yang et al. (2012), *Solanum lycopersicum TCP26* (Solyc03g045030.1), have consensus TCP-binding sites in their upstream non-coding sequences (Supplementary Table 4).

AtTCP1 binds to all combinations of the consensus sequence 5′-GGNCCC-3′ *in vitro*, and flanking regions have limited significance in this interaction (Gao et al., 2015). *In vivo*, AtTCP1 can directly bind to the two TCP-binding sites located in the regulatory region of the a downstream gene *DWARF4* (Gao et al., 2015). This suggests that the *Arabidopsis* TCP1 transcription factor can likely bind to the predicted TCP-binding site in its own upstream non-coding sequence, and hence possibly undergoes DTA. *AtTCP1* is expressed and is functional across the shoot organs throughout development, from seedlings to inflorescences (Koyama et al., 2010). This persistent expression is consistent with it having a predicted autoregulatory site. Expression surveys employing *in situ* mRNA hybridization (Cubas et al., 2001) and *AtTCP1* promoter fused to a β-glucuronidase (GUS) construct (Koyama et al., 2010) did not detect *AtTCP1* expression in later stages of flower development. It is interesting that the expression of a gene that is widely expressed in and controls development of many different organs is specifically downregulated in flowers. It is possible that *AtTCP1* is negatively regulated during late stages of *Arabidopsis* flower development, or continues to be expressed in flowers but a level that can only be detected by more sensitive methods, like quantitative rt-PCR.

Predicted *CYC2*-lineage autoregulatory sites are strongly associated with monosymmetry supporting the potential importance for DTA in establishing high and continuous asymmetric expression through later stages of flower organ differentiation (Figure 6). However, this pattern is not exclusive: *CYC2*-lineage orthologs from many species lacking monosymmetry also have predicted TCP-binding sites. This may be autoregulation for alternative developmental pathways, or regulation of *CYC2*-lineage genes by upstream TCP activators. At this point, experimental tests of TCP gene autoregulation are too sparse to draw solid conclusions regarding the role of DTA in independent origins of flower monosymmetry across core eudicots.

### Origin and Evolution of Autoregulatory Sites in DTA

Any *cis*-regulatory site can evolve by two primary processes, *de novo* by mutation and/or recombination in ancestral non-regulatory sequences, or by duplication of existing regulatory sites from a different location in the genome. Both have been reported in the origin of *cis*-regulatory sites involved in DTA. For example, the *CArG*-*box* sites involved in *Arabidopsis* AP1 autoregulation discussed earlier evolved by substitutions in the ancestral sequence that likely had a weak affinity for AP1 (Ye et al., 2016). Once evolved, these sites can undergo duplications, as reported in the apple MYB10 gene that controls fruit flesh color (Espley et al., 2009; van Nocker et al., 2012).

How did the predicted autoregulatory sites in *CYC2-*lineage genes originate? We did not detect consensus TCP-binding sites with accompanying flanking sequences elsewhere in the *A. majus* or *M. lewisii* genomes. This suggests that these predicted autoregulatory sites evolved *in situ* and are not a result of duplication from a different part of the genome, i.e., similar to the origin of the autoregulatory sites in *Arabidopsis* *AP1* (Ye et al., 2016). However, multiple consensus TCP-binding sites are present within single *A. majus* and *M. lewisii* *CYC2-*lineage genes. To further test whether these multiple TCP-binding sites within a single putative regulatory region evolved by local, intra-genic duplication, as in the case of *MYB10* promoter in apples (Espley et al., 2009; van Nocker et al., 2012), we aligned all *A. majus* and *M. lewisii* consensus TCP-binding sites, along with 100 bp flanking on either side, from within single upstream non-coding regions. We found no evidence that any of the predicted TCP-binding sites are derived from tandem duplication within *CYC* regulatory regions, again suggesting that multiple binding sites evolved *de novo.*

### Chicken or Egg: Novel Function or DTA First?

We have discussed potential roles of DTA in development, but how does DTA itself evolve? Autoregulation is common among genes positioned upstream in genetic regulatory networks with crucial developmental functions (discussed in Crews and Pearson, 2009; Hoot et al., 2010; specifically tested in yeasts and hepatocytes by Pasqualucci et al., 2003; Odom et al., 2006; Hervay et al., 2011; Tao et al., 2012). This observed pattern leads to an interesting chicken or egg conundrum. Which evolves first in genes recruited to new developmental functions: the novel function, or the autoregulation? Two scenarios can explain the observed pattern that crucial genes are often autoregulated. (1) DTA evolves first, and such genes are recruited for new functions that require extended stable expression. Or, (2) New function evolves first, and such genes, under selective pressure to provide extended stable expression, evolve DTA.

Evidence supporting scenario 2 is found in the *Arabidopsis* *AtAP1* example. This A-class floral homeotic gene in Brassicaceae underwent a duplication that generated the paralogs *AP1* and *CAL* gene lineages (Wang et al., 2012). AtAP1 defines sepals in *Arabidopsis thaliana*, but this function has not been reported elsewhere, and is likely an innovation in the genus *Arabidopsis* (Huijser et al., 1992; Lowman and Purugganan, 1999; Shepard and Purugganan, 2002; Litt, 2007; Ruokolainen et al., 2010). Except for the *AP1* paralog in *Arabidopsis* species, no Brassicaceae *AP1/CAL* gene tested to date undergoes DTA (Ye et al., 2016). And, as described above, DTA is an integral component of AtAP1 A-class function in flower development. Further, while the *AP1* orthologs of two *Arabidopsis* species have *CArG*-*box* in their *cis*-regulatory region that allows them to undergo DTA, other Brassicaceae species have *CArG-box*-like sequences with mismatches in the homologous gene region. In one such homolog, *Capsella rubella AP1*, the binding affinity of the mismatched *CArG*-*box*-like sequence was tested and can only weakly bind to AP1 protein. Hence, *Capsella rubella AP1* is likely not autoregulated (Ye et al., 2016). This suggests that the autoregulation of *Arabidopsis* *AP1* evolved either after or during, but not before, its recruitment to A-class function.

A major unanswered question that will clarify the origin of DTA in *Arabidopsis* *AP1* is whether its orthologs have similar functions in other Brassicaceae species. It is challenging to identify the ancestral state of autoregulation for any gene primarily for two reasons: there has been little functional work outside the model species, and predictive surveys are limited because genomes sequencing has been biased toward lineages with those model species. As plant sciences expands away from models systems (Poaceae, Brassicaceae, and Solanaceae), a wider phylogenetic sampling will facilitate reconstruction of ancestral molecular interactions.

## Conclusion

The origins and evolution of autoregulation will likely remain elusive until extensive experimental evidence emerges from multiple plant (and animal) lineages that inform ancestral and derived roles for autoregulation in development. It is, however, not surprising that a large number of transcription factors involved in defining crucial or novel phenotypes undergo DTA, as this form of regulation is expected to both enhance and stabilize gene expression patterns critical for developmental patterning. We find evidence for enrichment of self-binding sites in Lamiales *CYC2*-lineages genes. This enrichment may reflect evolution of a novel pattern of DTA early in Lamiales diversification, coincident with the origin of a key morphological innovation, floral monosymmetry. It is likely that the putative autoregulatory binding sites associated with Lamiales *CYC2*-lineages genes evolved via *de novo* mutations. Whether DTA is conserved across Lamiales awaits further experimental evidence, as does the hypothesis that independent origins of flower monosymmetry may be associated with the evolution of positive transcriptional autoregulation.

## Author Contributions

LH co-conceived of this project, oversaw analyses, and contributed to writing the manuscript. AS co-conceived of this project, carried out analyses, and contributed to writing the manuscript.

## Conflict of Interest Statement

The authors declare that the research was conducted in the absence of any commercial or financial relationships that could be construed as a potential conflict of interest.

## Footnotes

1 http://tree.bio.ed.ac.uk/software/figtree/

2 http://meme-suite.org/tools/ame
