# Supplementary material for: Novel Traits, Flower Symmetry, and Transcriptional Autoregulation: New Hypotheses From Bioinformatic and Experimental Data
Source: Front Plant Sci. 2018 Oct 26;9:1561. doi: 10.3389/fpls.2018.01561 (PMC6212560; doi:10.3389/fpls.2018.01561)
Supplement: Supplementary file 5 [file Image_1.pdf]

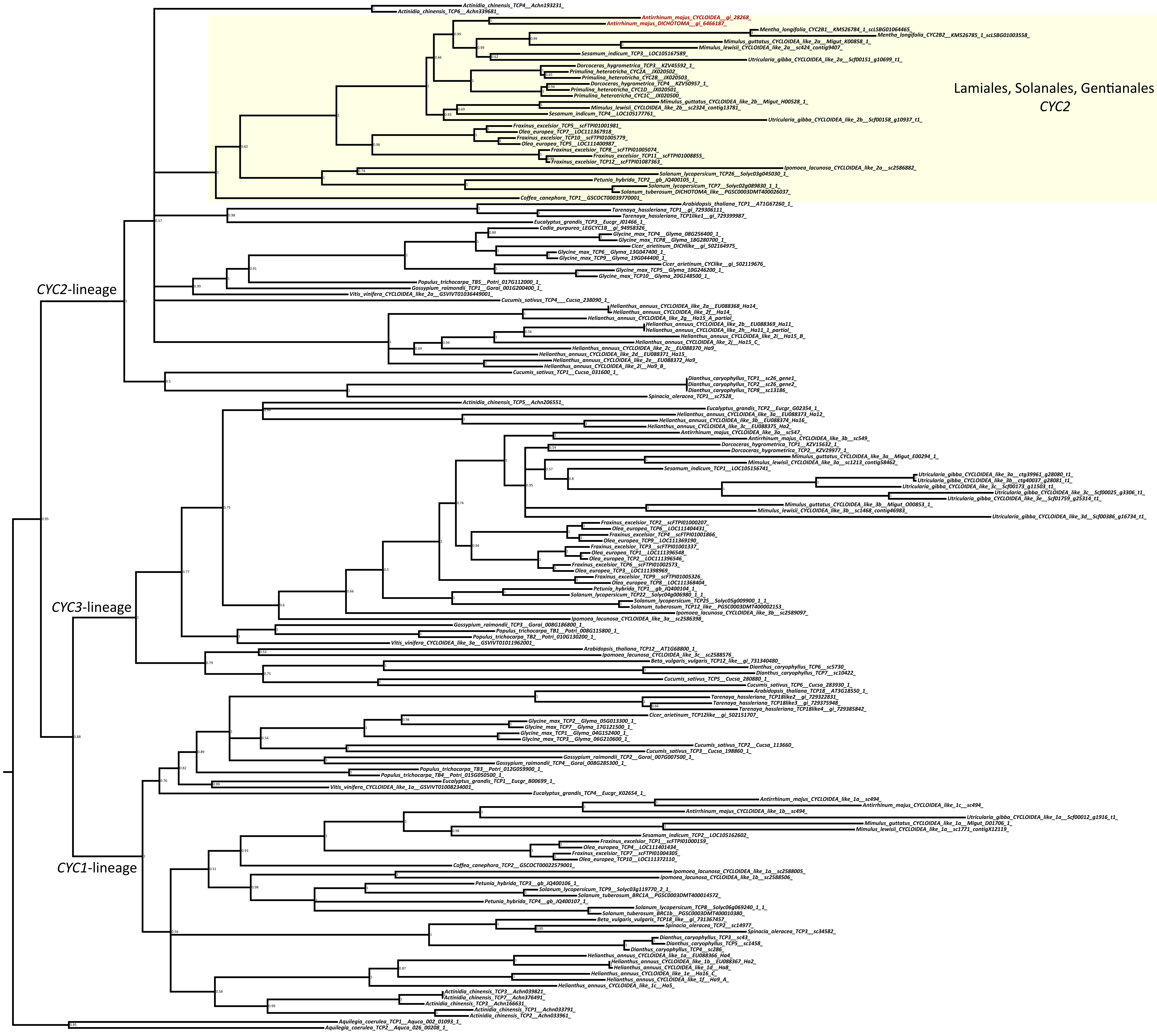

Supplementary Figure 1. Bayesian majority-rule consensus tree of *CYC*-like genes in eudicots. Bayesian posterior probabilities at nodes. Unique gene identifiers are indicated for each terminal. Additional information for identifiers can be found in Supplementary Table 1.
