# Supplementary material for: Novel Traits, Flower Symmetry, and Transcriptional Autoregulation: New Hypotheses From Bioinformatic and Experimental Data
Source: Front Plant Sci. 2018 Oct 26;9:1561. doi: 10.3389/fpls.2018.01561 (PMC6212560; doi:10.3389/fpls.2018.01561)
Supplement: Supplementary file 6 [file Image_2.PDF]

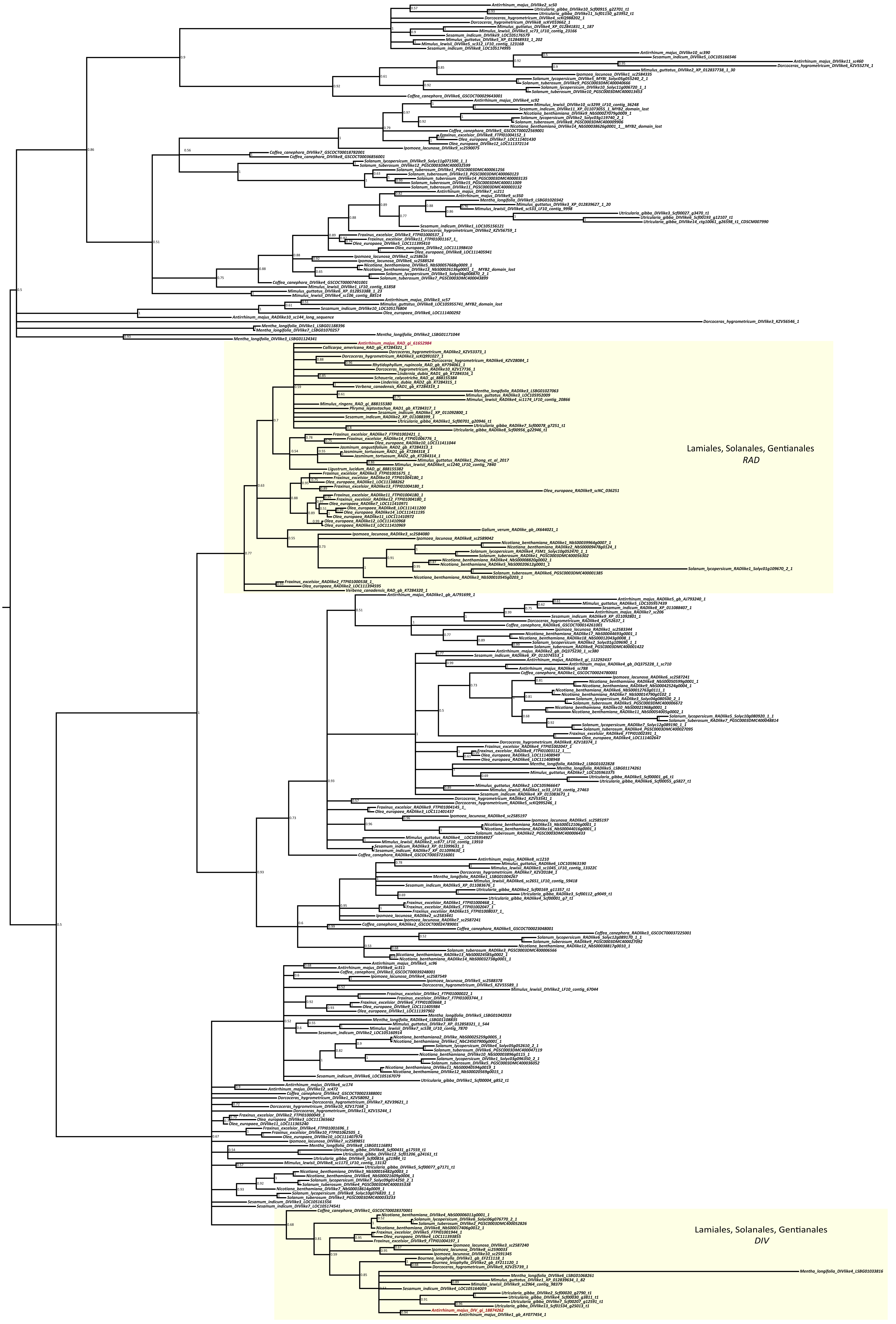

Supplementary Figure 2. Bayesian majority-rule consensus tree of *DIV-* and *RAD*-like genes in Lamiales, Solanales, and Gentianales. Bayesian posterior probabilities at nodes. Unique gene identifiers are indicated for each terminal. Additional information for identifiers can be found in Supplementary Table 2.
